# Flower-like Cu_2_SnS_3_ Nanostructure Materials with High Crystallinity for Sodium Storage

**DOI:** 10.3390/nano8070475

**Published:** 2018-06-28

**Authors:** Lin Fu, Zhen Bi, Benben Wei, Lanyan Huang, Xuzi Zhang, Zhihong Chen, Hua Liao, Ming Li, Chaoqun Shang, Xin Wang

**Affiliations:** 1 International Academy of Optoelectronics at Zhaoqing, South China Normal University, Zhaoqing 526060, China; fulin.work@foxmail.com (L.F.); chenzhihong1227@sina.com (Z.C.); 2 National Center for International Research on Green Optoelectronics, South China Normal University, Guangzhou 510006, China; zhen.bi@ecs-scnu.org (Z.B.); benben.wei@ecs-scnu.org (B.W.); lanyan.huang@zq-scnu.org (L.H.); xuzi.zhang@ecs-scnu.or (X.Z.); 3 Shenyang Institute of automation, Chinese Academy of Sciences, Guangzhou 511458, China; 4 Institute of Solar Energy, Yunnan Normal University, Kunming 650500, China; liaohua8@ynnu.edu.cn (H.L.); lmllldy@126.com (M.L.)

**Keywords:** Cu_2_SnS_3_, high crystallinity, anode material, sodium ion batteries

## Abstract

In this study, ternary Cu_2_SnS_3_ (CTS) nanostructure materials with high crystallinity were successfully prepared via a facile solvothermal method, which was followed by high-temperature treatment. The morphology of the as-synthesized samples is uniform flower-like spheres, with these spheres consisting of hierarchical nanosheets and possessing network features. Sodium storage measurements demonstrate that the annealed CTS electrodes have high initial reversible capacity (447.7 mAh·g^−1^ at a current density of 100 mA·g^−1^), good capacity retention (200.6 mAh·g^−1^ after 50 cycles at a current density of 100 mA·g^−1^) and considerable rate capability because of their high crystallinity and unique morphology. Such good performances indicate that the high crystallinity CTS is a promising anode material for sodium ion batteries.

## 1. Introduction

With the global energy crisis, it has become increasingly urgent to develop high-performance electrical energy storage devices, such as lithium ion battery (LIB), sodium ion battery (NIB) and supercapacitor [1,2,3,4,5]. Due to their high theoretical specific capacities and conductivity, ternary alloy-based sulfides have been extensively studied as anode materials for rechargeable energy storage batteries [6]. In particular, the intermediate products during charging/discharging processes can serve as inert matrices to accommodate volume changes that result from the alloy reactions and to mitigate agglomeration of active material particles [7]. Cu_2_SnS_3_ (CTS), which is an important semiconductor, has been used extensively because it has an optimal band gap (1 eV), good hall mobility (1.79 cm^2^·V^−1^·s^−1^) and high carrier concentration (1.85 × 10^20^ cm^−3^) [8,9,10]. Interestingly, the crystalline structure of CTS has interlayer spaces and tunnels with superior conducting ability, which indicates that CTS is a promising electrode material for rechargeable batteries [10]. In addition, introducing inactive Cu into host materials can improve their electrical conductivity and mitigate volume changes during the charging/discharging processes [11]. Nanostructured CTS and CTS/reduced graphene oxide composites have been used as anode materials for LIBs, with these materials exhibiting considerable cycling and rate properties [12,13].

CTS may be a promising candidate for NIBs because the fundamental characteristics of NIBs are similar to those of LIBs. Furthermore, a CTS supercell crystalline structure, which has a tunnel size of 3.921 Å × 5.587 Å × 4.210 Å and an interlayer distance of 2.280 Å, has been shown to be adequate for facilitating the diffusion of sodium ions, which have a radius of 1.12 Å [14,15]. Shi et al. successfully synthesized CTS nanosheets via a solution chemical method and explored the performance of the CTS nanosheets as anode materials for NIBs [16]. At a constant current density of 30 mA·g^−1^ for 50 cycles, CTS nanosheets exhibit an initial discharge capacity of 586 mAh·g^−1^, initial Coulombic efficiency (CE) of 59.5% and a discharge capacity that decays to 178 mAh·g^−1^. The electrochemical performance of crystalline materials is better than that of their amorphous counterparts [17]. We designed and prepared high-crystalline CTS by annealing the solvothermal products in an argon atmosphere to further improve the storage properties of NIBs. The as-synthesized CTS electrodes have a high initial reversible capacity (447.7 mAh·g^−1^) and favorable capacity retention at a current density of 100 mA·g^−1^. These results demonstrate that the rational design of nanostructures is a promising method for enhancing the electrochemical performance of CTS used in NIBs.

## 2. Materials and Methods

### 2.1. Preparation of Materials

A simple solvothermal method was used to synthesize CTS powders. Copper chloride dihydrate (2 mmol, CuCl_2_·2H_2_O), tin (IV) chloride pentahydrate (1 mmol, SnCl_4_·5H_2_O), thioacetamide (3 mmol) and polyethylene glycol (30 mL, PEG-200) were dissolved in a 200-mL beaker. The mixed solution was heated at 60 °C for 40 min and magnetically stirred to form a black emulsion. The emulsion was transferred into a 50-mL Teflon-lined stainless steel autoclave, which was heated at 180 °C for 16 h and cooled naturally to room temperature. Precipitates were separated via centrifugation and washed several times with deionized water and absolute alcohol to obtain a black powder, which was dried at 60 °C for 12 h in a vacuum oven. The as-prepared samples were annealed at 500 °C in an argon atmosphere for 4 h to improve crystallinity.

### 2.2. Characterizations of Materials 

The crystallinity and phase composition of the as-synthesized samples were characterized using an X-ray diffractometer (XRD, Bruker D8 advance, Karlsruhe, Germany) with Cu Kα radiation (λ = 1.5406 Å). The morphological characteristics of the samples were examined using a field emission scanning electron microscope (SEM, Hitachi S-4800, Tokyo, Japan). Transmission electron microscopy (TEM), high-resolution TEM (HRTEM) and energy dispersive X-ray (EDX) spectroscopy mapping images were recorded using a field emission transmission electron microscope (Tecnai F20 G^2^, Hillsboro, USA). The selected-area electronic diffraction (SAED) patterns were acquired using a JEM 2100HR TEM (Tokyo, Japan).

### 2.3. Electrochemical Measurements

To prepare CTS electrodes, 80 wt % CTS, 10 wt % conductive carbon (Super P) and 10 wt % polyacrylic acid binder were dispersed homogeneously in an appropriate amount of water. After this, the above slurry was coated on Cu foil and the electrodes were dried at 60 °C for 12 h in a vacuum to remove the water. Electrochemical performances were evaluated using 2016 coin-type cells with a glass microfiber filter as a separator and a Na metal counter/reference electrode. The electrolyte used was 1 mol NaClO_4_ in ethylene carbonate/diethyl carbonate (1:1 *v*/*v*) with 5% fluoroethylene carbonate as an additive. Cyclic voltammetry (CV) was performed using an electrochemical workstation (CHI 660E). CV was conducted at a scan rate of 0.2 mV·s^−1^ with a voltage range of 0.05–2.5 V vs. Na^+^/Na. Galvanostatic charge/discharge cycling measurements were obtained using a computer-controlled battery tester system (Land CT2001A) with the current densities of 50–500 mA·g^−1^ and with the voltage range of 0.05–2.5 V vs. Na^+^/Na.

## 3. Results and Discussion

**Figure 1:**
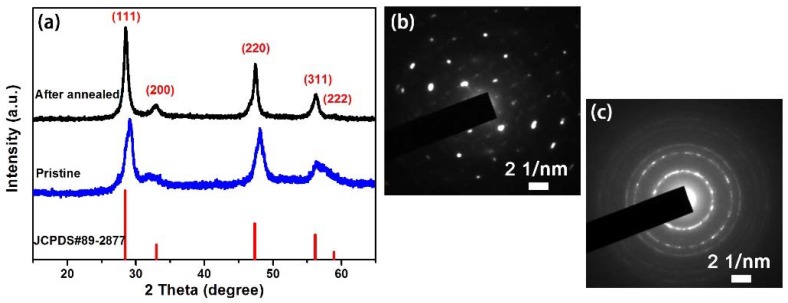
(**a**) XRD patterns of the pristine and annealed CTS. SAED patterns of the (**b**) annealed and (**c**) pristine CTS.

CTS nanomaterials were prepared via a facile solvothermal process, before being annealed at 500 °C for 4 h in an argon atmosphere to enhance crystallinity. As seen in Figure 1, all of the diffraction peaks were entirely consistent with the standard JCPDS card (No. 89-2877). No impurity peaks were found, which demonstrates that the as-prepared CTS products were highly pure. The average crystallite sizes of pristine and annealed CTS were 82.08 and 131.21 Å, respectively, by the Scherrer equation (*d* = *kλ*/(*βcosθ*), where *d* is the average crystal diameter, *k* is the shape factor, *λ* is the X-ray wavelength, *β* is the line broadening at half the maximum intensity (FWHM) and *θ* is the Bragg angle based on XRD data, which demonstrates that the calcination temperature of 500 °C can accelerate the further growth of the CTS crystallite structure [18,19]. More importantly, the diffraction peaks of the annealed CTS were stronger than those of the pristine CTS counterparts, which indicates that annealing improved the crystallinity. The SAED patterns of the annealed CTS (Figure 1b) further demonstrate better crystallinity compared with that of the pristine CTS (Figure 1c).

**Figure 2:**
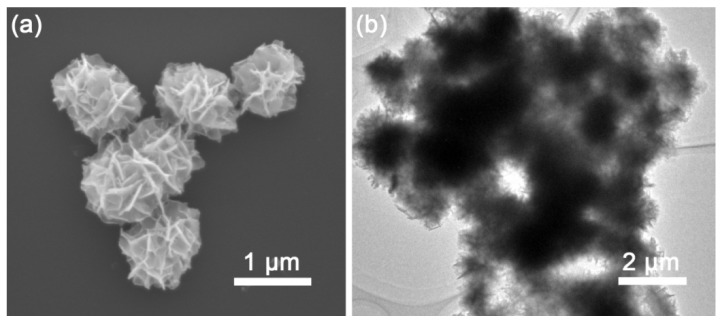
(**a**) SEM and (**b**) TEM images of the pristine CTS.

**Figure 3:**
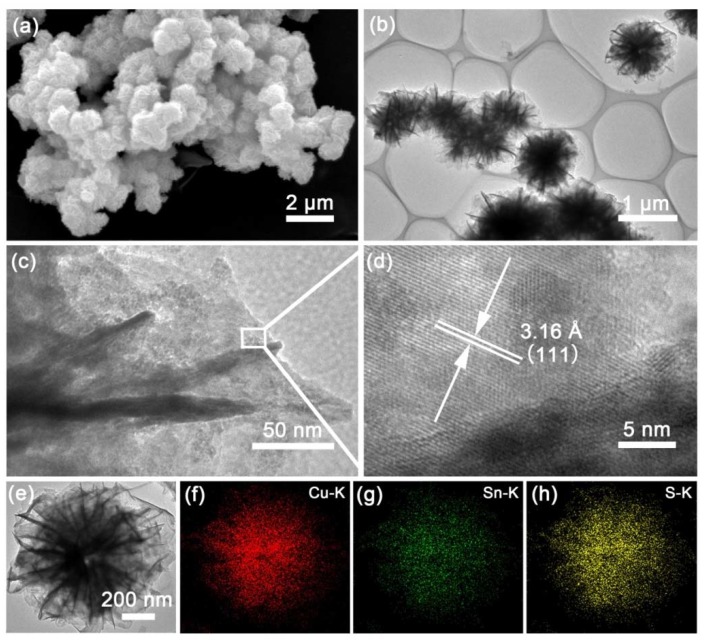
(**a**) SEM, (**b**,**c**) TEM and (**d**) HRTEM images of CTS. (**e**) TEM image and the corresponding EDX mapping of (**f**) Cu, (**g**) Sn and (**h**) S of the annealed CTS.

The morphological characteristics and microstructure of the as-synthesized samples were observed using field emission SEM and TEM. Figure 2a presents a typical SEM image of pristine CTS, which shows that it is composed of flower-like spheres that have uniform dimensions with a diameter of about 1 μm. These spheres consist of hierarchical nanosheets that are assembled into a flower-like structure at the surface edges. Figure 2b presents a TEM image of the unique flower-like spheres, which shows that they have network features. SEM (Figure 3a) and TEM (Figure 3b) images indicate that the annealed sample maintains the hierarchical flower-like microstructure, with this stable structure helping to achieve favorable cycling performance. Figure 3c shows a typical high-magnification TEM image of the annealed CTS and confirms that the spheres were formed from interconnected nanosheets. Figure 3d shows a HRTEM image of the lattice fringes and a corresponding interplanar spacing of 3.16 Å, which is ascribed to the (111) plane of the cubic CTS structure. These results are consistent with the XRD results and indicate that the as-synthesized materials have good crystallinity. A TEM image of individual flower-like spheres and corresponding EDX mapping images are shown in Figure 3e–h, which show that Cu, Sn and S were evenly distributed along the K-lines in the flower-like CTS spheres.

**Figure 4:**
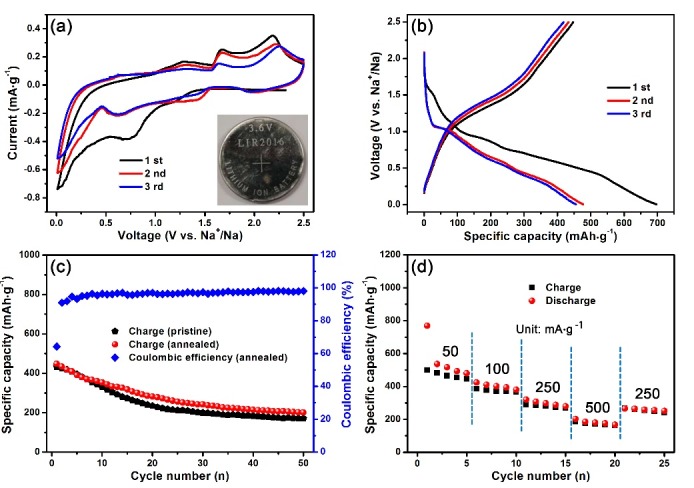
(**a**) CV curves for the first three cycles of annealed CTS at a scan rate of 0.2 mV·s^−1^. Inset in Figure 4a is the photo of 2016 coin type cell; (**b**) Charge/discharge curves of CTS at a current density of 100 mA·g^−1^; (**c**) Cycling performance of pristine and annealed CTS at a current density of 100 mA·g^−1^; (**d**) Rate capability of annealed CTS at various current densities of 50–500 mA·g^−1^.

The sodium storage properties of CTS were determined using the CTS/Na half-cell. Figure 4a shows the CV curves for the first three consecutive cycles of annealed CTS at a scan rate of 0.2 mV·s^−1^. In the cathodic scan, a weak peak at around 1.2 V and a sharp peak centered at 0.7 V were detected in the first cycle. These two peaks moved to 1.45 and 0.6 V, respectively, in both the second and third cycles. These peaks can be attributed to the multi-step decomposition of CTS with sodium ions to generate Cu, Sn and Na_x_S [12,20,21]. The oxidation peak at 0.7 V was stronger for the initial scan than the other sweeps because of the irreversible formation of a solid electrolyte interface (SEI) film [22]. A pair of oxidation redox peaks positioned at 0.05 V/0.6 V is attributed to the alloying/dealloying reaction of Sn with sodium [22,23]. In the anodic scan, a smooth peak at 1.32 V indicates the desodiation of Na_x_S [24]. In addition, the two sharp peaks found at about 1.68 and 2.25 V are associated with the regeneration of CTS [13,25]. Thus, the theoretical specific capacity of CTS is ca. 763.8 mAh·g^−1^ calculated for the formation of Cu, Na_2_S and Na_3.75_Sn [26]. The CV curves, especially in the first scan, are almost overlapping, which demonstrates the good reversibility of the CTS/Na half-cell during sodium insertion/extraction.

The electrochemical performance of CTS was explored using galvanostatic charge/discharge measurements. Figure 4b shows the first three charge/discharge profiles at a current density of 100 mA·g^−1^. The voltage plateaus are consistent with CV results. In addition, the initial charge and discharge capacities of the annealed CTS electrode were 447.7 mAh·g^−1^ and 696.8 mAh·g^−1^, respectively, which correspond to an initial CE of 64.25%. The capacity loss of 35.75% can be mainly attributed to the SEI film formation because of the large surface of the flower-like structure. As displayed in Figure 4c, the pristine CTS delivered an initial charge capacity of 433.2 mAh·g^−1^ and a residual capacity of 170 mAh·g^−1^ after 50 cycles at a current density of 100 mA·g^−1^, which is close to the previously reported CTS anode materials used for sodium storage [16]. It is interesting that the annealed CTS also presented a high reversible capacity of 200.6 mAh·g^−1^ after 50 cycles at the same current density (4.8 mAh·g^−1^ capacity loss for per cycle from the first cycle to 50th cycle). Because of its good crystallinity, which is the result of the high-temperature treatment, the electrochemical performance of the annealed CTS electrode was better than that of pristine CTS. The annealed CTS electrodes were cycled five times at 50, 100, 250 and 500 mA·g^−1^, before being cycled at 250 mA·g^−1^ for five final cycles to evaluate their rate properties (Figure 4d). Reversible capacities of 464, 370, 279 and 172 mAh·g^−1^ were recorded at the current densities of 50, 100, 250 and 500 mA·g^−1^, respectively. When the current density was returned to 250 mA·g^−1^, the charge specific capacity remained at 254 mAh·g^−1^, which was 91.04% of its capacity at the same current density. This good rate performance might be associated with the good conductivity of the crystalline material.

## 4. Conclusions

Flower-like CTS spheres with high crystallinity that have uniform dimensions were successfully synthesized via a facile solvothermal reaction, which was followed by high-temperature treatment. The XRD and SAED results demonstrate that the crystallinity of annealed CTS is higher than those of the pristine counterparts. Surprisingly, it is found that the morphology of the annealed CTS was well-maintained compared with that of before annealing. This high stable structure is beneficial for achieving favorable electrochemical performance for alloy-based materials. When the annealed CTS is used as an anode material for NIBs, it exhibits considerable electrochemical properties, including high initial CE (64.25%), good capacity retention (200.6 mAh·g^−1^ after 50 cycles at a current density of 100 mA·g^−1^) and favorable rate capability. These properties can be ascribed to the high crystallinity and unique morphology of CTS. These results indicate that CTS with high crystallinity is a promising anode material for NIBs.
